# A case of Ewing's sarcoma identified via noninvasive prenatal testing

**DOI:** 10.1002/ccr3.2673

**Published:** 2020-03-14

**Authors:** Keiko Miyagami, Ryu Matsuoka, Mayumi Tokunaka, Nahoko Shirato, Mikiko Izumi, Tatsuko Hirose, Akihiko Sekizawa

**Affiliations:** ^1^ Department of Obstetrics and Gynecology Showa University School of Medicine Tokyo Japan

**Keywords:** cell‐free DNA, incidental finding, malignant tumor, NIPT, prenatal diagnosis

## Abstract

Although noninvasive prenatal testing is not intended to identify maternal genomic information, it can provide other information that may lead to the incidental discovery of coexisting conditions including maternal malignancy.

## Graphical abstract

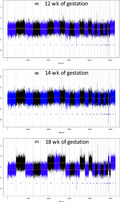

## 1 INTRODUCTION

Noninvasive prenatal testing (NIPT) through the analysis of plasma cell‐free DNA (cfDNA) has been available as a commercial test since 2011. In Japan, NIPT of trisomies 21, 18, and 13 has become available since April 2013 as a nationwide clinical trial. Over 60 000 tests were performed by the Japan NIPT consortium in the first 6 years since its introduction, with more than 90% of cases analyzed via massively parallel sequencing (s‐MPS) for the DNA analyses. Among the pregnant women tested, 1.8%, 97.9%, and 0.3% obtained positive, negative, and inconclusive results, respectively.^1^ The positive predictive value of trisomy 21 was over 96%, and the negative predictive value of three trisomies was <1 in 10 000.^1^ Previously, we conducted NIPT analysis of cfDNA in maternal plasma comprising maternal and placental DNA, and the median fetal fraction of samples having euploid results was 13.7%.^2^ Therefore, some factors, including cotwin demise, confined placental mosaicism, maternal chromosomal mosaicism, variations in maternal DNA copy number, maternal transplant from a donor, and maternal malignancy, might affect the result of NIPT. Herein, we present a case of Ewing's sarcoma in the pelvic cavity that was incidentally detected through the nonreportable result of NIPT.

## 2 CASE PRESENTATION

**Figure 1:**
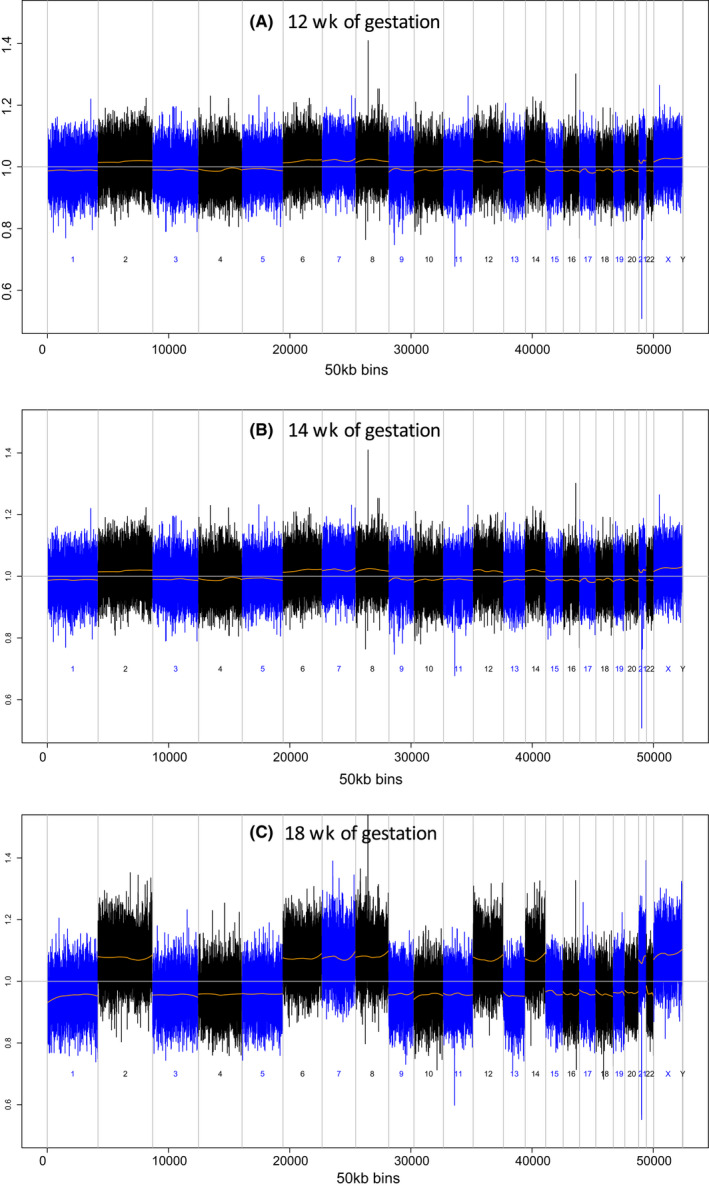
Whole genome view of copy number gains and losses in plasma of pregnant woman. In the NIPT at 12, 14, and 18 wks of gestation, the copy number gains were observed in chromosome 2, 6, 7, 8, 12, 14, and 21. The CNAs were enhanced during the pregnancy according to the growth of the tumor

A 35‐year‐old gravida 3 para 1 woman presented to our hospital at 12 weeks of gestation with no remarkable prior medical history except for thyroid tumor. Ultrasound examination revealed that her ovary and uterus were normal and that the fetus had normal growth and did not have any significant abnormality. She requested for NIPT after precise genetic counseling, and NIPT was performed at 12 weeks of gestation. Because the fraction of fetal cfDNA was approximately 12%, and multiple fetal aneuploidies were suspected (Figure 1A), the result was classified as nonreportable. The patient was informed of the result during genetic counseling at 14 weeks of gestation, and invasive testing such as chorionic villous sampling or amniocentesis was offered, but she refused invasive testing and requested for a repeat NIPT instead. However, the result of the second NIPT was still “nonreportable,” suggesting multiple aneuploidies in the fetus (Figure 1B). Ultrasound examination at 14 and 16 weeks of gestation revealed normal fetal, placental, ovarian, and uterine structure without any myomas and tumors in the pelvic cavity. She was counseled about the “nonreportable” NIPT result and the advantage of amniocentesis to determine fetal aneuploidy, but the patient still refused an invasive test and opted to follow the natural course of pregnancy. A thyroid disease specialist was consulted due to her history of thyroid tumor, and her thyroid size and function were normal. Therefore, her pregnancy was managed in an outside clinic.

At 18 weeks of gestation, she visited our hospital for a third NIPT. However, although similar result was obtained, the Z‐scores of the corresponding chromosomes were markedly increasing (Figure 1C). Abdominal ultrasonography did not show any tumor in the abdominal cavity. Moreover, tumor marker tests, such as that for CA19‐9 7.4 U/ml, CEA 1.0 ng/ml, sIJ‐2R 242 U/ml, showed normal findings.

**Figure 2:**
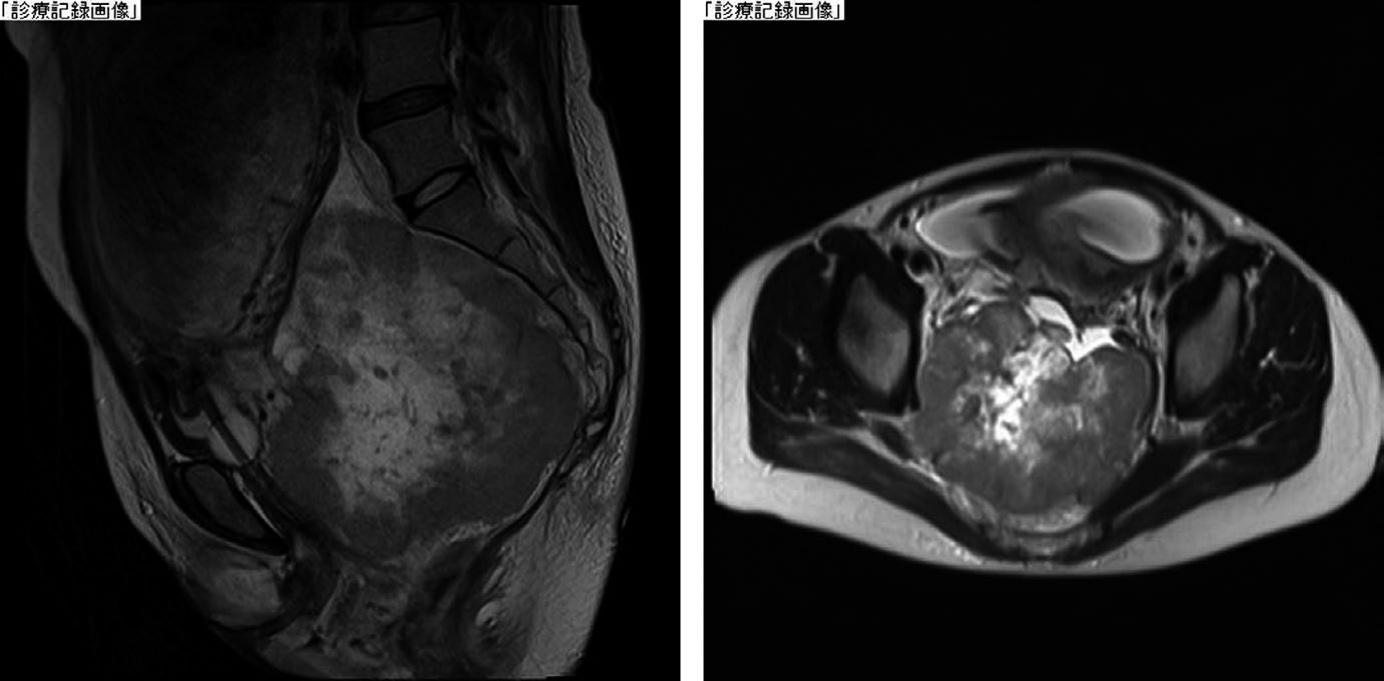
MRI image showed an 11 × 12 cm solid mass in the pelvis at 25 wks of gestation

**Figure 3:**
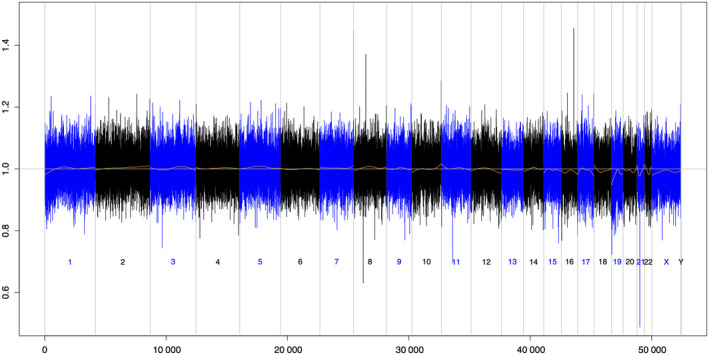
After the chemotherapy, abnormal CNAs were no longer detected

At 25 weeks of gestation, she visited the outside clinic due to an obstinate constipation. Transvaginal ultrasound after internal examination revealed pelvic tumor of 10 cm in diameter. Thereafter, we evaluated the tumor via pelvic magnetic resonance imaging (MRI), and an 11 × 12 cm solid mass was detected in the pelvis. The tumor showed similar characteristics to that of a leiomyosarcoma or gastrointestinal stromal tumor (Figure 2). It was unclear whether the tumor originated from the sacral bone or has invaded into the sacral bone. At 27 weeks of gestation, she was hospitalized because of severe abdominal pain. Imaging showed the tumor was rapidly increasing in size. Transvaginal biopsy of the tumor showed “malignant neoplasm.” Because the patient developed numbness and severe pain of the legs, cesarean section was performed at 29 weeks of gestation. The patient gave birth to a normal female infant weighing 1231 g with an Apgar score 6/10 (1 min/5 min). Chromosomal analyses revealed that the karyotypes of the infant and placenta were normal (46, XX). Intraoperative biopsy was performed, and the pathological examination revealed that the tumor was Ewing's sarcoma. Thereafter, the patient was treated for her malignancy through chemotherapy starting from 8 days after childbirth. At 10 months postpartum and after the chemotherapy, NIPT was performed again. Abnormal copy number alterations (CNAs) were no longer detected, and the result supported other clinical findings that the tumor in the pelvis has already disappeared (Figure 3).

The recurrence was not detected 24 months postpartum. Informed consent was obtained from the patient to report the clinical course.

## 3 DISCUSSION

Because NIPT analyzes the cell‐free DNA in maternal plasma, the result is affected by the maternal pathology. In cases in which the test result is discordant with the fetal status, further assessments should be performed to determine the underlying biological conditions, such as confined placental mosaicism, a twin demise, and maternal malignancy. Cancer rarely coexists with pregnancy, with an incidence of approximately 1 in 1000 pregnant women.^3^ The most common types of malignancy during pregnancy are Hodgkin and non‐Hodgkin lymphoma; breast, ovarian, and cervical cancers; malignant melanoma; colorectal cancer; and leukemia. Tumor DNA is believed to shed directly into the maternal circulation, and the tumor‐derived DNA is detectable in maternal plasma when the malignant tumor DNA contains multiple areas of duplications and deletions across the genome. Benign tumors such as uterine leiomyomas also shed DNA into the bloodstream.^4^ This leads to unusual multiple aneuploidies that would not be expected in a living fetus, which in turn result in nonreportable findings in NIPT and a suspicion of complicating malignancy in the pregnant woman.

Bianchi et al^5^ reported that 20%‐44% of discordant NIPT results that included multiple aneuploidies could potentially be explained by maternal malignancies. Dharajiya et al^6^ reported that an abnormal genomic profile in the plasma of pregnant women that is inconsistent with fetal abnormalities was detected in approximately 1 out of 10 000 cases. In their series, there were 43 cases with nonreportable NIPT in which follow‐up survey was possible, and 18 of these cases were attributed to maternal malignant neoplasms. A recent study using sequencing to analyze plasma cfDNA in patients with known cancers found evidence of abnormal cfDNA patterns in more than 80% of metastatic solid tumor cases and 50% of localized cancers.^7^ Our previous study also demonstrated that the incidence of copy number alterations (CNAs) is higher in advanced ovarian and uterine endometrial cancer than that in the corresponding early cancer; moreover, progression‐free survival and overall survival in cases with CNAs were shorter than those without CNAs.^8^ Thus, CNAs could be detected in a definite proportion of pregnant women with malignancies, and the alteration might affect the accuracy of NIPT.

**Figure 4:**
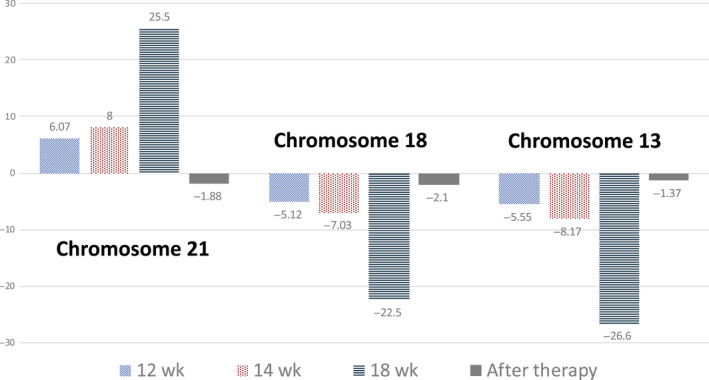
Z‐score of each chromosome in NIPT

In the present case, NIPT result indicated multiple aneuploidies that would not be expected in a living fetus, leading to a nonreportable test result at 12 and 14 weeks of gestation. Although no abnormal mass was detected in the abdominal cavity on ultrasound examination at 14 and 16 weeks of gestation, repeated NIPT showed increasing Z‐score in multiple chromosomes, and the alterations were believed to have reflected further tumor growth. The initial detection of the alteration at 12 weeks of gestation was limited, but it was markedly enhanced at 14 weeks and then 18 weeks in a stepwise manner as shown in Figure 4. The tumor grew rapidly, and this affected the increase of the DNA alteration. This dynamic alteration indicated that the proportion of altered genome profiles in the plasma might be a potential factor for estimating disease progression. In the third NIPT at 18 weeks, the Z‐score was considerably higher than the first and second Z‐score, indicating that the malignancy needed more attention. If MRI was performed during the earlier gestation stage, the tumor might have been detected. In the study by Amant et al,^9^ 3 of more than 4000 pregnant women undergoing NIPT had aberrant genome representation profiles in their plasma. Through the use of whole‐body MRI, an ovarian carcinoma, a follicular lymphoma, and Hodgkin's lymphoma were found, but they reported that performing whole‐body MRI for all nonreportable results of NIPT is not ideal because of the high cost. However, MRI may be useful for detecting the mass in cases with abnormal NIPT results; thus, we believe that MRI should be considered an option for detecting the origin of CNAs in cases with multiple aneuploidies in NIPT while the fetus appears normal.

In a position statement of the American College of Medical Genetics and Genomics, they recommend that patients and providers should be aware of the potential for inadvertent discovery of other information during NIPT, including maternal malignancy, and the potential for additional follow‐up testing unrelated to the pregnancy, although NIPT is not intended to identify clinically relevant maternal genomic information.^10^ The present case shows the importance of pretest genetic counseling for pregnant women to understand the potential for incidental diagnosis of maternal disorders.

In conclusion, we experienced a case of nonreportable NIPT result that was affected by Ewing's sarcoma during pregnancy. The CNAs were enhanced during the pregnancy according to the growth of the tumor, and the alterations disappeared after the chemotherapy. These findings indicated that CNAs are a potential marker for detecting and monitoring the existence and growth of the tumor. Furthermore, the case shows that the presence of maternal malignant tumor should be considered when abnormal NIPT result is obtained. Moreover, repeat NIPT or MRI should be considered for the differential diagnosis of malignancy. Clinicians should be adequately knowledgeable about the implications of NIPT findings and should inform pregnant women about the potential for incidental findings during pretest genetic counseling.

## CONFLICT OF INTEREST

We do not have any conflict of interest.

## AUTHOR CONTRIBUTIONS

KM and RM: wrote this report. MT and NS: examined the patient. MI and TH: provided genetic counseling. AS: oversaw the production of the manuscript. All authors: managed the patient in the clinical setting and contributed equally. All authors: read and approved the final manuscript.
